# Harmfulness Score: A Data‐Driven Framework for Ranking Environmental Risks of Microplastics

**DOI:** 10.1002/marc.202500559

**Published:** 2025-10-21

**Authors:** Fernando Gomes Souza, Shekhar Bhansali, Thomas Thundat

**Affiliations:** ^1^ Instituto De Macromoléculas Professora Eloisa Mano Universidade Federal do Rio de Janeiro Centro de Tecnologia–Cidade Universitária Rio de Janeiro Brazil; ^2^ Programa de Engenharia da Nanotecnologia – COPPE Universidade Federal do Rio de Janeiro Centro de Tecnologia–Cidade Universitária Rio de Janeiro Brazil; ^3^ Department of Electrical and Computer Engineering Florida International University Miami Florida USA; ^4^ Dept. of Electrical and Computer Engineering Vanderbilt University Nashville Tennessee USA; ^5^ Department of Chemical and Biological Engineering University At Buffalo–The State University of New York Buffalo New York USA

**Keywords:** harmfulness Score, microplastics, polymer risk assessment, machine learning, regulatory science

## Abstract

The analysis of 104,471 scientific abstracts on microplastics and nanoplastics using bibliometric tools and machine learning models produced a comprehensive mapping of thematic trends and material‐specific risk associations. A composite Harmfulness Score was constructed by integrating sentiment analysis, impact descriptors, and network centrality metrics. This score ranked polystyrene (PS) and polyethylene (PE) highest in association with terms such as oxidative stress, cytotoxicity, and genotoxicity, reflecting their prominence in the literature. Reporting frequencies for key physicochemical descriptors were low—particle size (3.91%), density (0.01%), and surface area (<0.01%)—limiting their use in computational modeling and risk assessments. Thematic clustering revealed dominant topics such as environmental policy and biological impact, alongside emerging areas in microbial degradation, enzymatic transformation, and legal‐policy intersections. The results highlight the need for standardized metadata practices and expanded use of analytical frameworks to enhance research reproducibility and policy relevance.

## Graphical abstract

Using machine learning and bibliometric sentiment analysis on over 100 000 abstracts, this study introduces the Harmfulness Score to rank environmental risks of microplastic polymers. Polystyrene and polyethylene emerge as top‐risk materials, supporting targeted regulation and evidence‐based policy recommendations.

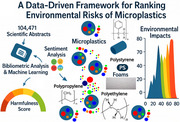

## 1 Introduction

Microplastics (<5 mm) and nanoplastics (<1 µm) [1, 2, 3] are persistent environmental pollutants that adsorb hazardous substances such as heavy metals and persistent organic pollutants [4], triggering oxidative stress, inflammation, and gene expression alterations in exposed organisms, with potential toxic effects on human tissues [5, 6].

However, regulatory efforts remain fragmented, hampered by inconsistent sampling protocols, scarce reporting of key particle traits like size, surface area, and density, and the absence of standardized evaluation metrics [7]. Global mitigation is further impaired by the lack of clear, science‐based thresholds for identifying and classifying harmful polymers, leaving regulatory decisions vulnerable to political negotiation rather than scientific rigor [8].

Leveraging natural language processing and machine learning, we introduce the Harmfulness Score, a composite index derived from sentiment polarity, impact‐related term frequency, and network centrality metrics, applied to over 100 000 peer‐reviewed studies [9]. This model reveals critical patterns, identifies hazardous polymers such as polystyrene and polyethylene, highlights underreported descriptors, and clarifies the role of particle size and polymer–nanoparticle interactions in environmental risk. By merging computational analysis with toxicological evidence, this scalable strategy supports regulatory prioritization and advances coordinated global responses to microplastic pollution (see Supporting Information).

## 2 Results and Discussion

### 2.1 Bibliometric Mapping and Thematic Clustering of Microplastic Research

**FIGURE 1:**
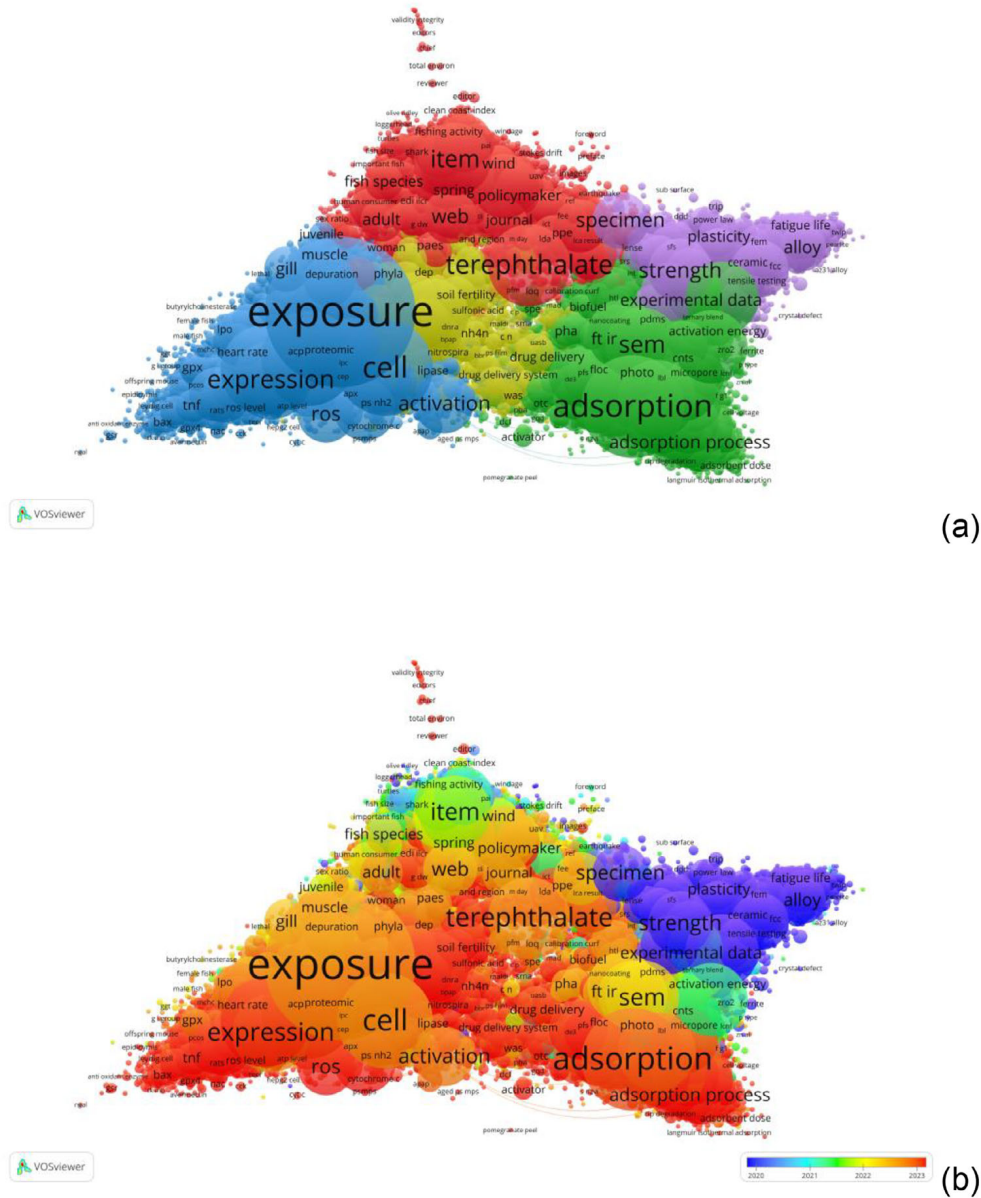
Co‐occurrence network of terms in microplastic and nanoplastic research from 1961 to 2025, generated using VOSviewer. (a) Network visualization map showing clustered thematic areas based on term co‐occurrence frequency and total link strength. (b) Overlay visualization map displaying the average publication year of terms, highlighting temporal trends and emerging research topics.

The bibliometric analysis of 104 471 documents revealed a strong correlation between term frequency and Total Link Strength (R^2^ = 0.953), indicating that frequently mentioned terms such as exposure, oxidative stress, and adsorption are also the most structurally central in the co‐occurrence network (Figure 1a). Seven thematic clusters were identified: environmental policy (Cluster 1), adsorption and materials (Cluster 2), biological and toxicological effects (Cluster 3), microbial interactions (Cluster 4), mechanical characterization (Cluster 5), legal and publishing aspects (Cluster 6), and niche studies involving pesticide–microplastic interactions (Cluster 7).

Temporal overlay analysis (Figure 1b) showed that terms introduced in 2023–2024 are concentrated in interdisciplinary areas, including artificial intelligence applications in Cluster **1** (e.g., large language model, RMSE value), advanced functional materials in Cluster **2** (e.g., piezoelectric catalysis), systems toxicology in Cluster **3** (e.g., lipophagy, panoptosis), and metagenomic tools in Cluster 4. Term relationship analysis confirmed the conceptual centrality of toxicological terms, with the strongest links observed between exposure and oxidative stress (LSBI = 1761), exposure and gene (LSBI = 1501), and expression and gene (LSBI = 1391), while adsorption and adsorption capacity (LSBI = 1200) reflected terminological precision in materials research.

### 2.2 Automated Data Extraction and Variable Frequency Analysis From Titles and Abstracts

The metadata analysis revealed that environmental impact was the most frequently cited variable, appearing in 73 708 abstracts (82.85%), followed by characterization techniques in 27 628 (31.06%), chemical composition in 21 881 (24.60%), polymer type in 21 714 (24.41%), and particle morphology in 18 609 (20.92%). Mentions of engineered nanoparticles were found in only 6427 abstracts (7.22%). Reporting of key physicochemical descriptors was minimal, with particle size mentioned in 3481 abstracts (3.91%), density in 13 (0.01%), and surface area in just 4 entries (<0.01%), limiting their integration into environmental models and toxicity assessments. The low frequency of nanomaterial terms and inconsistent documentation of composition and morphology highlight a lack of standardization in metadata reporting across the literature.

### 2.3 Particle Size Distributions and Morphological Trends in Microplastic Characterization

Figure 2 shows an integrated visual analysis of microplastics. The analysis of microplastic particle sizes identified a multimodal distribution ranging from 0 to 5 mm, modeled through nonlinear regression using a four‐curve composite envelope, achieving a high fit accuracy (WSSR = 0.0074; R^2^ = 0.9997; df = 992), as shown in Figure 2a.

FIGURE 2Integrated visual analysis of microplastics: Probability density distribution of particle sizes, highlighting multimodal peaks with scaled representations of dominant diameters (a); morphological distribution of microplastic shapes cross‐referenced by polymer type, revealing prevalent fragmentation patterns (b); co‐occurrence heatmap of polymer and engineered nanoparticle (ENP) associations in microplastics research, indicating functional pairings and environmental relevance (c).

**Figure:**
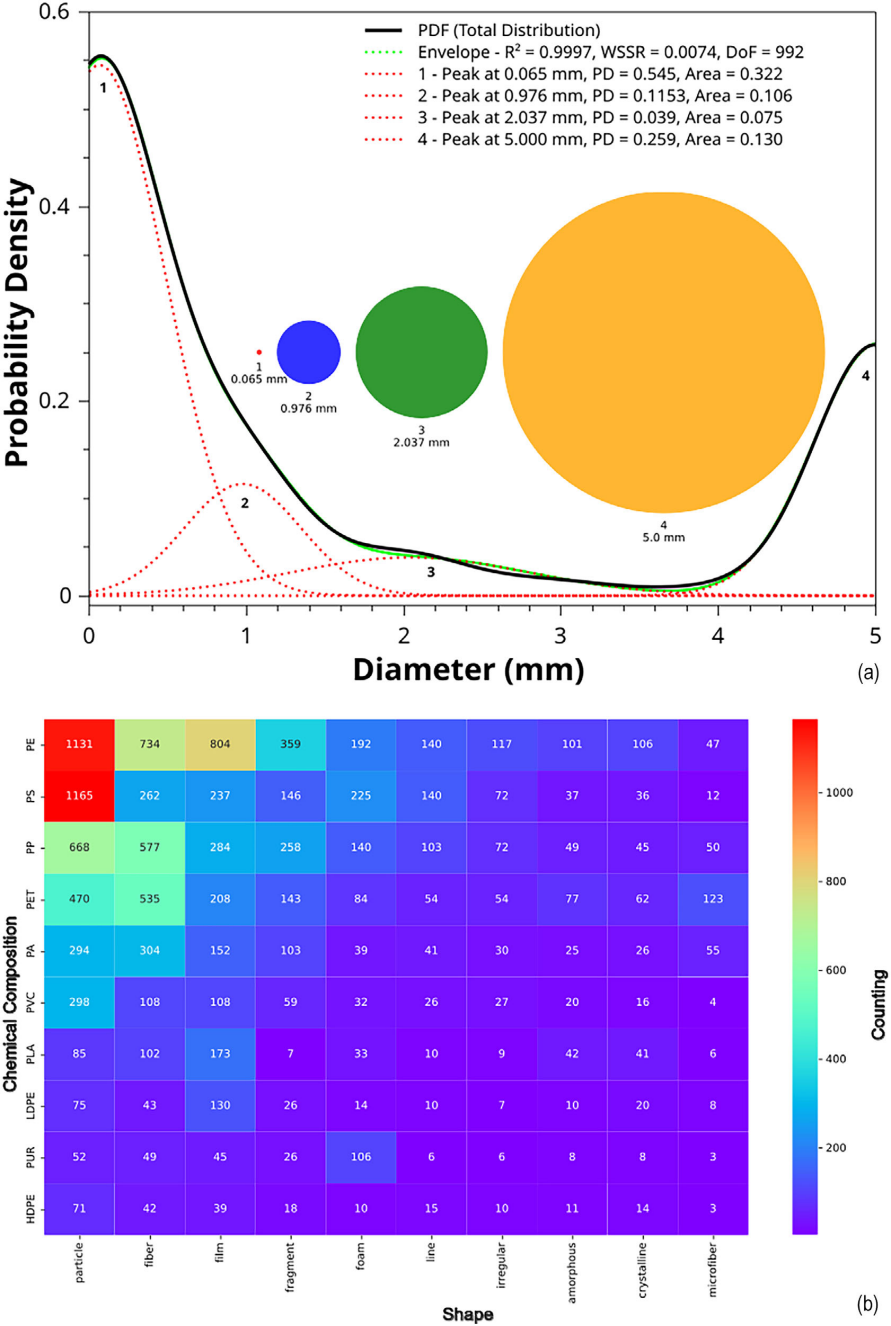

**Figure:**
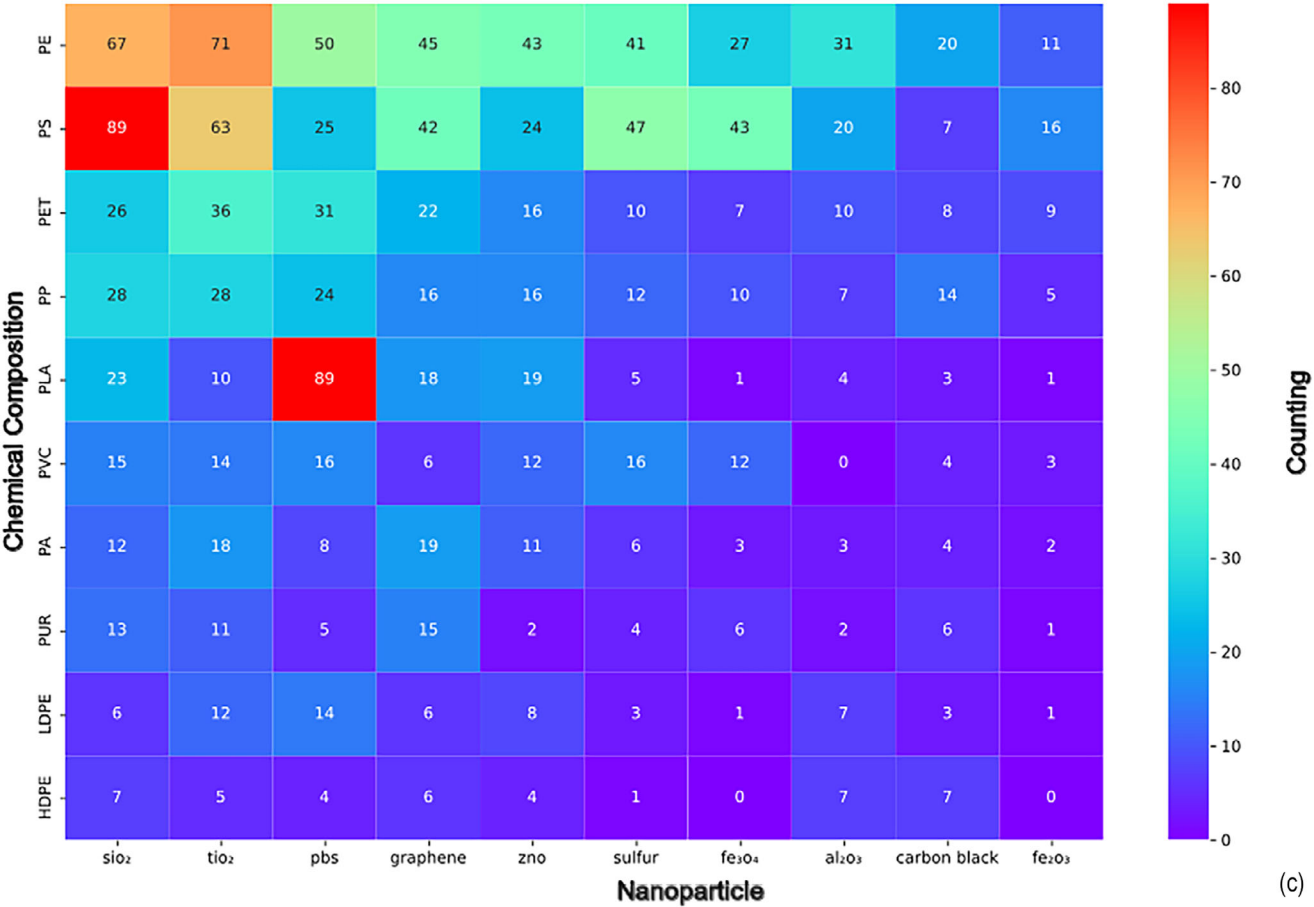

Curve 1 peaked at 0.065 mm and accounted for 32.2% of the integrated probability, indicating a dominant presence of fine particles bordering the nanoplastic scale. Curves 2 to 4 captured sub‐millimeter (0.976 mm) and larger fragments (2.037 mm and 5 mm) [10, 11]. The high density of particles near the lower limit aligns with bibliometric findings showing that 6.38% of the literature refers to nanoparticle interactions, reinforcing relevance to the nano–microplastic continuum [12, 13]. These results build on prior studies such as Koelmans et al. 2020 [14], by addressing earlier inconsistencies through a combined modeling and semantic framework. The particle size data were also integrated into a composite Harmfulness Score based on clustering, sentiment, and network centrality, supporting ecological risk assessments. Despite this integration, polymer density and surface area remained largely unreported in abstracts, confirming persistent metadata gaps.

### 2.4 Polymer Compositions, Shapes, and Associations

The analysis of polymer compositions, shapes, and associations reveals that FTIR (3857) and SEM (3817) are the most frequently employed techniques, followed by Raman spectroscopy (2372), pyrolysis (2204), XRD (1849), fluorescence microscopy (2023), and digestion protocols (1975), with a notable rise in machine learning (2,998), PCA (774), and neural networks (535), indicating increasing automation in microplastic characterization workflows [15, 16, 17, 18].

Polymer prevalence in the literature aligns with global production and environmental persistence, with polyethylene (PE, 10 247) and polystyrene (PS, 7633) leading, followed by polypropylene (PP, 5579), PET (4609), PVC (2416), PA (2408), and biodegradable PLA (1507), while variants like LDPE (1,022) and HDPE (800) reflect differentiated applications, and rarer polymers such as PEEK appear only 22 times, confirming a literature focus on conventional thermoplastics [19].

Morphological descriptors extracted show “particle” as the most common term (6692), followed by “film” (3219), “fiber” (2718), “line” (1482), and others such as “fragment,” “foam,” and “crystalline,” while shapes like “sphere” (272), “bead” (183), and “pellet” (265) signal preproduction or cosmetic sources [20, 21].

Heatmap data in Figure 2b confirm that PE and PS dominate in “particle” morphology (1,131 and 1,165), with PE also prominent as “film” (804) and “fiber” (734), and PS notable in “foam” (225), “film” (237), and “fiber” (262), reflecting typical fragmentation pathways. PP exhibits high frequencies of “particle” (668), “fiber” (577), and “film” (280), consistent with its presence in textiles and packaging. PET, associated with synthetic fabrics, shows significant “fiber” (535), “particle” (470), and “film” (207) frequencies, while PA is fiber‐dominant (254 fibers, 50 microfibers), and PVC displays balanced morphology across forms.

PLA, though biodegradable, is represented by 173 films, 93 fibers, and 85 particles, indicating environmental persistence. LDPE and HDPE show distributions typical of films and rigid particles, while PUR is distinguished by its “foam” form (106). Microfibers are especially prevalent in PET (49), PA (50), and PP (46), reinforcing their role in aquatic microfiber pollution. Less common polymers like ABS, PC, and PMMA appear mostly as particles and fibers due to mechanical breakdown, and EVA, PTFE, and engineering polymers such as POM, PBT, and PEEK present consistent but infrequent shape signatures linked to their specific uses.

### 2.5 Polymer and Nanoparticles Associations

The associations between polymers and engineered nanoparticles (ENPs), visualized in Figure 2c, reveal a high frequency of co‐mentions that underscore both environmental and functional research interests.

Polyethylene (PE) exhibited the strongest associations, particularly with Titanium Dioxide (TiO_2_, 121), followed by Zinc Oxide (ZnO, 53), Silicon Dioxide (SiO_2_, 47), Silver Nanoparticles (AgNPs, 35), Iron Oxide (Fe_3_O_4_, 28), Graphene Oxide (GO, 20), Carbon Nanotubes (CNTs, 18), and Copper Oxide (CuO, 15). Polystyrene (PS) showed similarly high co‐occurrence with TiO_2_ (97), AgNPs (44), ZnO (30), SiO_2_ (25), and Fe_3_O_4_ (20). Polypropylene (PP) and PET followed comparable patterns, with TiO_2_ (62 and 55), ZnO (35 and 27), and other ENPs, including GO and CNTs. Polyamide (PA) and PVC both co‐occurred with TiO_2_, ZnO, and AgNPs, while PLA, a biodegradable polymer, appeared most frequently with TiO_2_ (26), AgNPs (17), GO (10), and CNTs (9). Across the dataset, TiO_2_ emerged as the most frequently paired ENP, linked to its role in UV protection, photocatalysis, and plastic degradation. ZnO and AgNPs were commonly associated with antimicrobial applications, while SiO_2_, GO, CNTs, and Fe_3_O_4_ reflected enhancements in mechanical, electrical, and magnetic properties. Notably, pairings involving PE and PS with TiO_2_, AgNPs, and Fe_3_O_4_ dominate in ecotoxicological studies, simulating pollutant interactions and combined toxicity [22, 23].

Figure 2c, therefore, documents the integration of functional nanomaterials with prevalent polymers and identifies how co‐deployment strategies extend from commercial applications to environmental studies. These polymer–nanoparticle associations serve as a reference framework for assessing environmental exposure scenarios, guiding green nanocomposite development, and supporting regulatory science on micro‐ and nanoplastics [23, 24].

These associations are not merely bibliometric but translate into functional and toxicological consequences. For example, polyethylene paired with TiO_2_ is often studied under UV conditions, where photocatalytic ROS production enhances toxicity, while polystyrene–AgNP composites have been shown to intensify oxidative stress and cytotoxicity in aquatic organisms. Such polymer–nanoparticle synergies were captured in our network centrality metrics and contributed to the Harmfulness Score, highlighting that environmental risk is shaped not only by the polymer itself but also by its interactions with engineered nanomaterials.

Quantitative evidence further substantiates these interactions. Laboratory studies have demonstrated that microplastics adsorb nanoparticles like ZnO, leading to chemical transformations (ZnO → Zn‐sulfide, Zn‐phosphate) that increase bioreactivity and persistence [25]. Combined exposures of MPs and AgNPs enhanced toxicity in marine ciliates compared to single‐pollutant exposures [26]. Meta‐analysis of MP–NP–pollutant interactions in microalgae revealed predominantly additive toxicity, with some antagonistic outcomes depending on conditions [27]. Laboratory findings also document intestinal and neural toxicity from polystyrene nanoplastics in zebrafish [28, 29] and complex pollutant‐vector effects [30, 31]. Field studies, though less frequent, confirm widespread co‐occurrence of MPs and nanoparticles in aquatic and sedimentary systems [32, 33], while modeling efforts predict co‐migration and combined exposure scenarios [34, 35]. Despite these insights, long‐term quantitative assessments remain scarce, leaving gaps in understanding cumulative risks, bioaccumulation, and biomagnification [29, 36]. Together, these findings [25, 26, 27, 28, 29, 30, 31, 32, 33, 34, 35, 36, 37, 38, 39, 40, 41] support the assertion that MP–NP interactions heighten environmental risks, but also underscore the need for standardized long‐term studies integrating laboratory, field, and modeling approaches.

### 2.6 Environmental and Biological Impact

**FIGURE 3:**
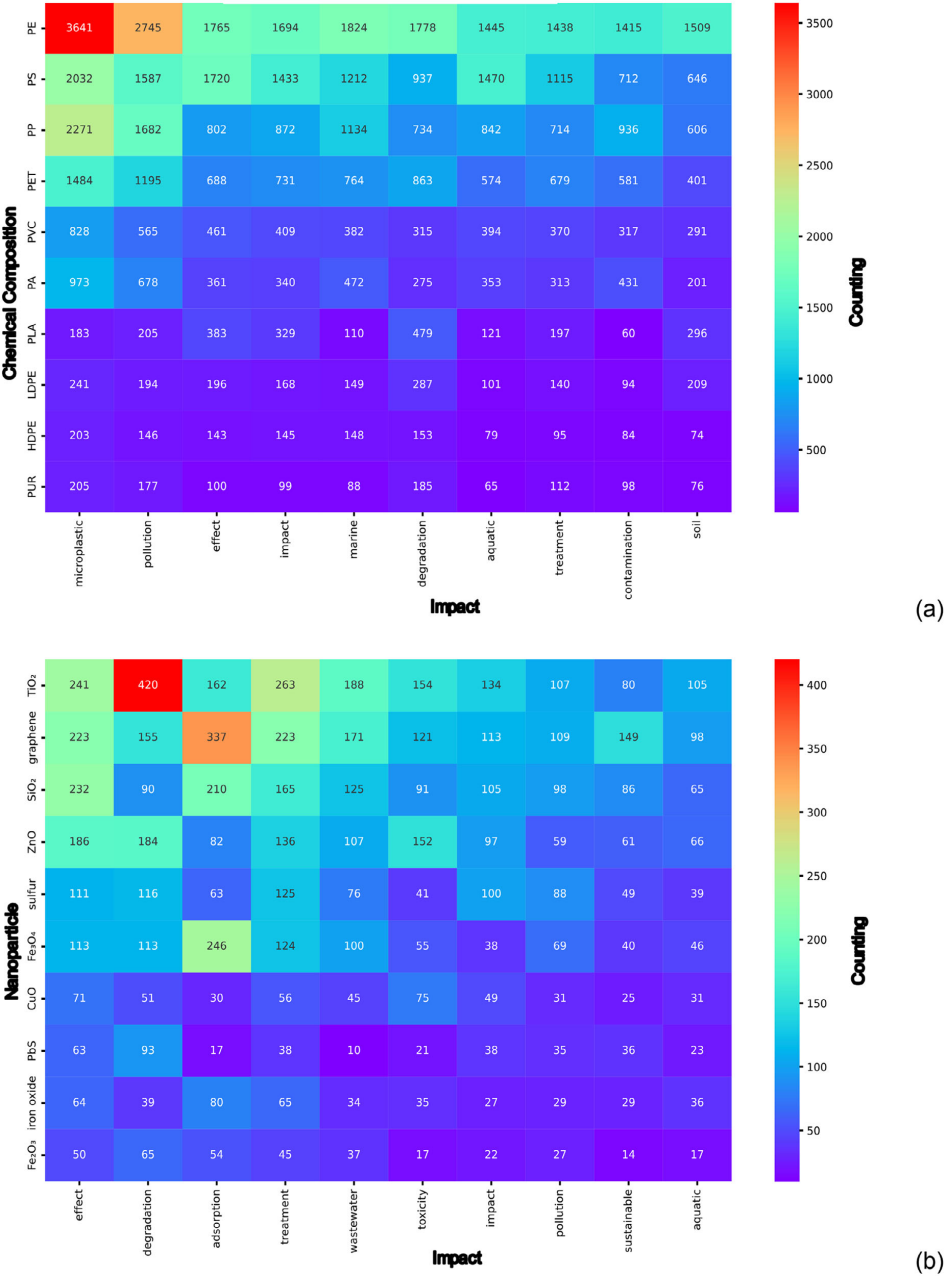
Heatmaps of Polymer and Nanoparticle Co‐Occurrences with Environmental and Biological Impact Descriptors. Co‐occurrence frequency between ten commonly studied polymers (Y‐axis) and ten impact categories (X‐axis) extracted from microplastics‐related scientific abstracts (a). Co‐occurrence frequency between ten engineered nanoparticles (Y‐axis) and ten impact categories (X‐axis) within the same corpus (b).

Microplastics and nanoplastics (MNPs) contribute to the carbon footprint indirectly by weakening ecosystem carbon sinks and amplifying greenhouse gas (GHG) fluxes rather than serving as direct fossil carbon sources. They alter soil microbial activity and biogeochemistry, affecting enzymes such as catalase and dehydrogenase and reshaping microbial community composition, which promotes respiration and reduces carbon sequestration [42, 43]. At the plant level, oxidative stress disrupts photosynthesis and gene expression, lowering carbon fixation efficiency and reprogramming metabolism toward defense pathways [43, 44, 45], while microbial gene expression changes further exacerbate GHG emissions [46]. In soils and aquatic systems, MNPs modify matrices by altering density, aggregation, and water‐holding capacity, impairing nutrient dynamics and fostering biofilm “hotspots” that accelerate respiration and suppress photosynthesis [43, 47, 48]. Their impacts extend to blue‐carbon ecosystems such as seagrasses, mangroves, and tidal marshes, where adsorption and uptake by photoautotrophs reduce carbon fixation and disrupt trophic chains, undermining the climate mitigation capacity of these critical sinks [45, 49, 50, 51]. The ubiquity of MNPs across soils, waters, and the atmosphere further amplifies these stresses [51]. Degradation processes release dissolved organic carbon that stimulates microbial respiration and CO_2_ emissions, while experimental studies confirm that MNPs also emit CO_2_, CH_4_, and N_2_O depending on polymer type and environmental conditions [44, 45, 46]. Although photo‐oxidative pathways are well documented, methane and ethylene release from sunlit plastics is not quantified in these three sources, representing an important research gap [43, 50]. Altogether, current evidence demonstrates that MNPs disrupt microbial and plant processes, alter environmental matrices, weaken blue‐carbon ecosystems, and enhance GHG emissions, thereby indirectly magnifying the global carbon footprint [43, 48, 50, 51, 52]. Consistent with these mechanisms, Figure 3 shows the heatmaps of polymer and nanoparticle co‐occurrences with environmental and biological impact descriptors.

Figure 3a presents a heatmap of co‐occurrence frequencies between ten polymers and ten environmental or biological impact descriptors in microplastics‐related literature. Polyethylene (PE) shows the highest values across all categories, including “microplastic” (3,641), “pollution” (2,745), and “effect” (1,765). Polystyrene (PS) follows with high frequencies in “microplastic” (2,032), “effect” (1,720), and “pollution” (1,587). Polypropylene (PP) also registers prominently in “microplastic” (2,271), “pollution” (1,682), and “impact” (872). Polyethylene terephthalate (PET) and polyvinyl chloride (PVC) appear frequently in “pollution”, “effect”, and “impact” categories, with PET recording 1,484 in “microplastic” and PVC 828. Polylactic acid (PLA) and polyamide (PA) show elevated frequencies in “degradation” (PLA: 479; PA: 353) and “effect” (PLA: 383; PA: 361). Lower frequencies are observed for LDPE, HDPE, and PUR, with LDPE and HDPE showing modest representation across all categories, and PUR being most frequently linked to “degradation” (185) and “treatment” (112).

Figure 3b shows co‐occurrence frequencies between ten engineered nanoparticles (ENPs) and ten impact categories. Titanium dioxide (TiO_2_) has the highest frequencies, including “degradation” (420), “effect” (241), “treatment” (263), “adsorption” (162), and “wastewater” (188). Graphene is prominent in “adsorption” (337), “effect” (223), “treatment” (223), and “sustainable” (149). Silicon dioxide (SiO_2_) shows high frequencies in “adsorption” (210), “treatment” (165), and “toxicity” (105). Zinc oxide (ZnO) is represented across all categories, with notable values in “effect” (186), “degradation” (184), “treatment” (136), and “toxicity” (152). Iron oxide (Fe_3_O_4_) is most associated with “adsorption” (246), while sulfur, copper oxide (CuO), lead sulfide (PbS), and iron oxides (Fe_2_O_3_, Fe_3_O_4_) show moderate frequencies across “toxicity”, “pollution”, and “treatment” categories. The data indicate varying degrees of representation across materials, with TiO_2_, graphene, and ZnO showing the highest co‐mention frequencies in multiple impact categories.

### 2.7 Geographic or Environmental Distribution of Studies

Figure 4 shows the country‐level distribution of polymer and nanoparticle mentions in microplastics‐related scientific literature.

FIGURE 4Country‐Level Distribution of Polymer and Nanoparticle Mentions in Microplastics‐Related Scientific Literature. Heatmap showing the number of co‐occurrences between ten countries (Y‐axis) and ten polymers (X‐axis): polyethylene (PE), polystyrene (PS), polypropylene (PP), polyethylene terephthalate (PET), polyamide (PA), polyvinyl chloride (PVC), polylactic acid (PLA), low‐density polyethylene (LDPE), high‐density polyethylene (HDPE), and polyurethane (PUR) (a). Choropleth map of the summed number of microplastic‐related mentions per country, aggregated from peer‐reviewed abstracts in Scopus (1961–2025) (b). Heatmap showing the number of co‐occurrences between ten countries (Y‐axis) and ten engineered nanoparticles (X‐axis): titanium dioxide (TiO_2_), graphene, silicon dioxide (SiO_2_), zinc oxide (ZnO), sulfur, iron oxide (Fe_3_O_4_), aluminum oxide (Al_2_O_3_), copper oxide (CuO), lead sulfide (PbS), and general iron oxide (c). Choropleth map of the summed number of nanoparticle‐related mentions per country in the context of microplastic studies (d).

**Figure:**
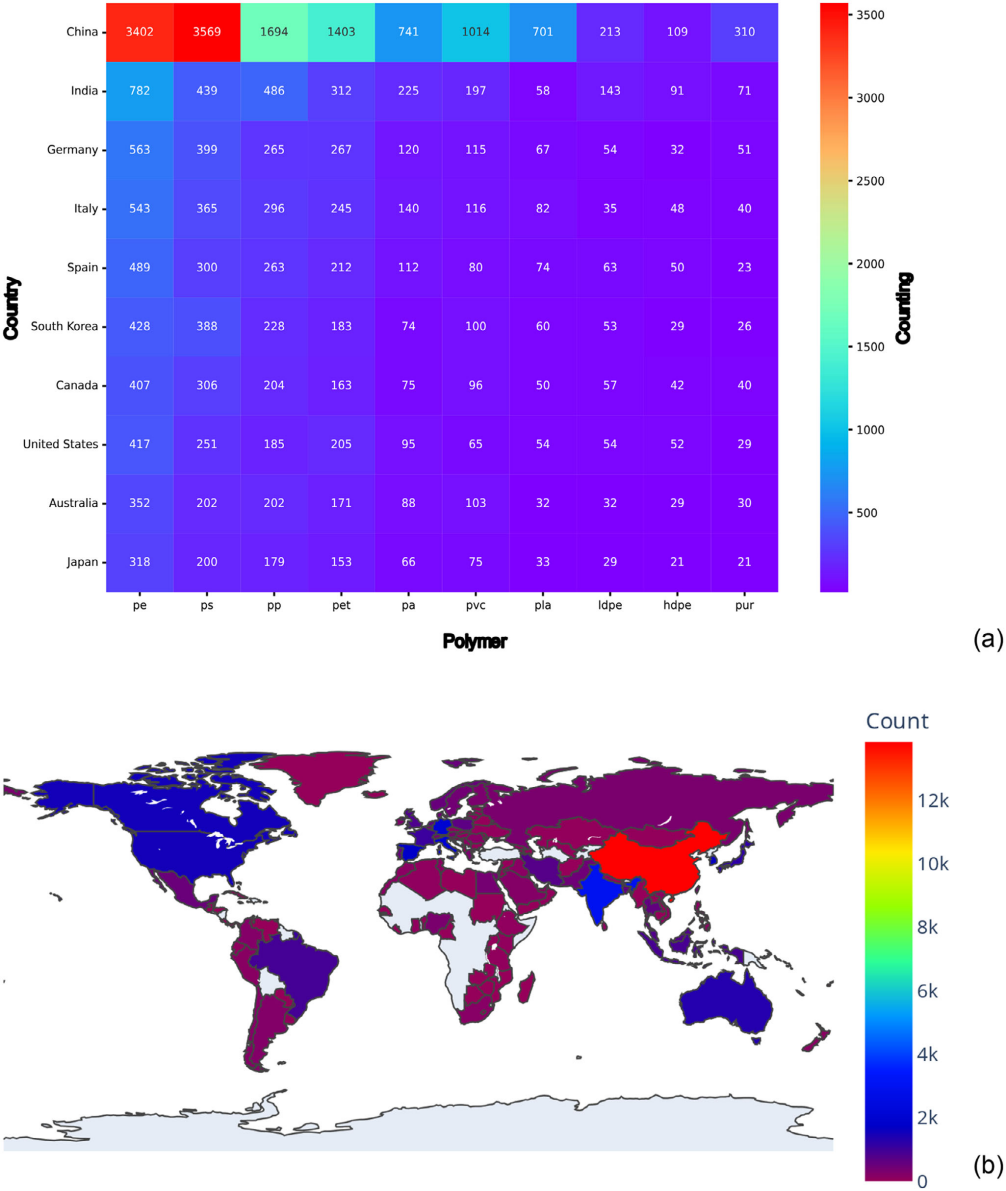

**Figure:**
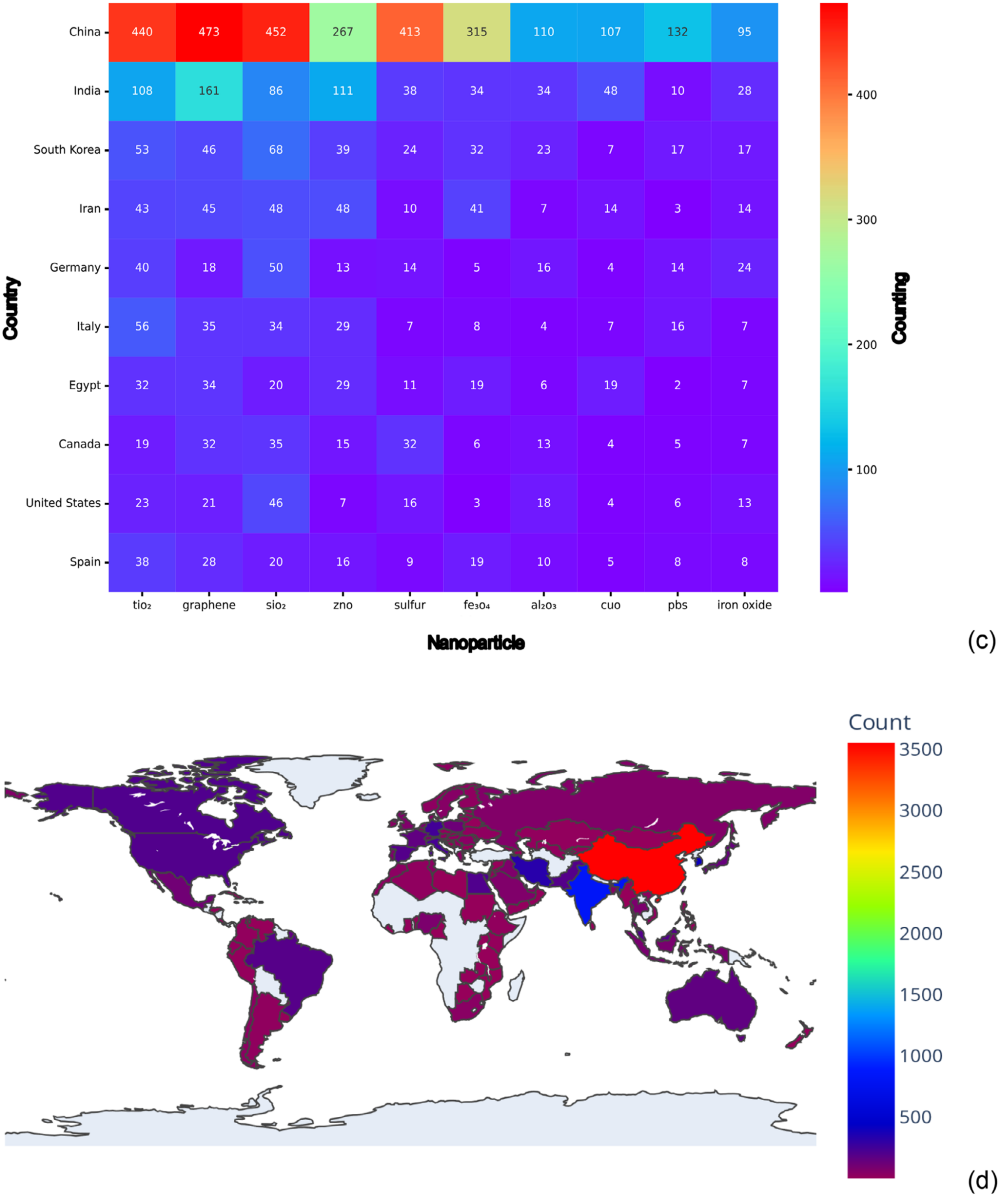

In Figure 4a, China records the highest co‐occurrence counts across all ten polymers, including PS (3,569), PE (3,402), PP (1,694), PET (1,403), PVC (1,014), and PLA (213). India follows with moderate counts, such as PE (782), PS (439), and PP (486). Germany, Italy, and Spain present intermediate frequencies, for example, Germany with 399 mentions each for PE and PS. South Korea, Canada, and the United States show consistent but lower counts, primarily in PE, PS, and PP. Australia and Japan register the lowest frequencies overall, with Japan showing a minimum of 21 mentions for PUR.

In Figure 4c, China again leads in mentions across all nanoparticles, including graphene (473), SiO_2_ (452), TiO_2_ (440), Fe_3_O_4_ (413), sulfur (315), ZnO (267), PbS (132), Al_2_O_3_ (110), CuO (107), and iron oxide (95). India ranks second, particularly for SiO_2_ (86), ZnO (111), and TiO_2_ (108). South Korea, Iran, and Germany contribute moderate counts, especially for TiO_2_, graphene, SiO_2_, and ZnO. Italy, Egypt, Canada, the United States, and Spain show lower frequencies, generally under 50 for each nanoparticle.

Figure 4b,d visually summarizes total counts by country. In both choropleth maps, China displays the highest cumulative values, followed by India and several countries in Europe and East Asia. The global distribution indicates concentration of polymer and nanoparticle research within the context of microplastics in a limited set of high‐output nations.

### 2.8 Machine Learning‐Based Harmfulness Assessment of Microplastic Polymers

Recent advances show that machine learning can both monitor and optimize the removal of microplastics (MPs) and nanoplastics (NPs) from water bodies. Hosseinzadeh et al. (2025) [53] modeled coagulation efficiency using 405 experimental datasets, showing that radial basis function networks achieved the highest accuracy (R^2^ = 0.96) and identified temperature and coagulant aids as key factors. Complementing this, Xie et al. (2025) [54] reviewed 111 studies applying **ML** to Raman and **IR** spectroscopy, demonstrating that algorithms such as Random Forest, **SVM**, and **CNN** can classify MPs and NPs with up to 98.8% accuracy, while also noting persistent challenges in dataset quality and environmental validation. However, none of these approaches provides a method to systematically rank the harmfulness of polymers, a gap that our work addresses by introducing a machine learning–based composite metric derived directly from literature metadata.

The Harmfulness Score was developed as a machine learning‐based composite metric to assess the environmental and toxicological risks posed by microplastic polymers. Based on 100 713 peer‐reviewed publications, two predictive pipelines using Random Forest and XGBoost were applied, incorporating sentiment analysis (VADER), environmental impact keyword frequencies, and network centrality measures derived from co‐occurrence networks. XGBoost demonstrated superior performance in sentiment prediction (accuracy = 0.699 vs 0.645), year estimation, and thematic clustering (Silhouette Score = 0.521; Davies‐Bouldin Index = 1.528), despite slightly higher **MSE** (18.003) and RMSE (4.243) compared to Random Forest (MSE = 17.617; RMSE = 4.197). These results support the adoption of XGBoost as the core model in Harmfulness Score computation.

The corpus underwent extensive preprocessing, including BoW and TF‐IDF vectorization, dimensionality reduction via TruncatedSVD (from 39 669 to 100 components), and KMeans clustering into 14 groups. Text normalization and sentiment polarity labeling revealed 65.5% positive, 24.2% negative, and 10.4% neutral tones. Degree centrality was used to quantify node importance due to eigenvalue convergence challenges in eigenvector centrality. This preprocessing facilitated the clustering and modeling steps, enabling the extraction of nuanced relationships between polymer types, environmental effects, and toxicity perceptions.

The Harmfulness Score was constructed as a composite index integrating three normalized layers of evidence. First, sentiment polarity from titles and abstracts was quantified using an enhanced VADER classifier with adjusted thresholds (compound ≥ 0.25 for positive, ≤ –0.25 for negative), yielding balanced distributions even under >50% neutral dominance. Second, the frequency of harmful impact descriptors was extracted through TF‐IDF vectorization and dimensionality reduction (TruncatedSVD, 100 components), ensuring that highly weighted terms reflected consistent toxicity signals rather than noise. Third, polymer–nanoparticle associations were captured through network analysis, where degree, betweenness, and eigenvector centralities highlighted entities strongly embedded in toxicological contexts. These components were aggregated into a base score and corrected by (i) a frequency weight that adjusted for publication imbalance across polymers and (ii) a confidence function penalizing underrepresented cases. Validation incorporated bootstrap resampling and model‐based robustness checks: optimized XGBoost regressors achieved stable R^2^ values (train ≈0.82, test ≈0.75) and low RMSE, while distributional analysis confirmed moderate skewness (≈1.75) and high internal consistency. The resulting values in Table 1 therefore reflect a harmonized integration of textual sentiment, toxicological intensity, and bibliometric centrality, robust to sampling variance and data imbalance. Further details on the calculations are provided in the Supplementary Information. The Harmfulness Scores for the 18 most prevalent polymers are shown in Table 1.

**TABLE 1:** Harmfulness Scores and contributing metrics for the 18 most prevalent microplastic polymers. The final score aggregates normalized sentiment, impact, and network centrality, weighted by frequency and confidence as defined in the methods.

Polymer	Harmfulness score	Base score	Frequency weight	Confidence	Sentiment component	Impact component	Centrality component	Paper count	Frequency percentage	Eigenvector centrality	Betweenness centrality
PS	0.790	0.857	0.744	0.997	0.770	0.874	1	7594	7.540	0.910	0.720
PE	0.771	0.772	1	0.998	0.737	0.694	1	10205	10.133	0.880	0.690
PP	0.663	0.771	0.545	0.996	0.709	0.719	1	5560	5.521	0.790	0.650
PET	0.629	0.757	0.449	0.996	0.748	0.644	1	4582	4.550	0.740	0.590
PA	0.618	0.809	0.236	0.992	0.804	0.718	1	2403	2.386	0.690	0.520
PVC	0.608	0.796	0.236	0.992	0.756	0.734	1	2405	2.388	0.680	0.510
PUR	0.598	0.848	0.076	0.975	0.942	0.679	1	780	0.775	0.590	0.480
PLA	0.573	0.781	0.147	0.987	0.974	0.478	1	1497	1.486	0.550	0.450
PC	0.568	0.809	0.070	0.973	0.828	0.696	1	718	0.713	0.520	0.430
PMMA	0.556	0.824	0.037	0.949	0.829	0.731	1	374	0.371	0.510	0.410
ABS	0.531	0.799	0.030	0.938	0.749	0.747	1	301	0.299	0.480	0.380
PTFE	0.520	0.804	0.021	0.916	0.864	0.730	1	217	0.216	0.450	0.350
EVA	0.519	0.850	0.013	0.868	0.708	1	1	131	0.130	0.440	0.330
HDPE	0.503	0.712	0.078	0.976	0.683	0.598	1	796	0.790	0.430	0.320
LDPE	0.495	0.692	0.100	0.981	0.682	0.548	1	1019	1.012	0.420	0.310
POM	0.484	0.775	0.015	0.887	1	0.438	1	157	0.156	0.380	0.280
PBT	0.468	0.831	0.008	0.802	0.791	0.869	1	81	0.080	0.360	0.250
PEEK	0.229	0.638	0.002	0.512	0.972	0.456	1	21	0.021	0.190	0.120

Among the polymers evaluated, polystyrene (PS) ranked as the most harmful (Score = 0.7895), owing to its widespread association with toxicity and its documented impacts on aquatic species, reproductive health, neurotoxicity, and inflammation across diverse organisms, including humans [55]. Polyethylene (PE), with a Harmfulness Score of 0.7709, was confirmed as a ubiquitous pollutant, exhibiting toxic effects in soil fauna, aquatic vertebrates, plants, and mammals, while also acting as a vector for organic pollutants under specific environmental conditions [56]. Polypropylene (PP) ranked moderately (Score = 0.6634), but emerging studies highlight its impacts on fish, mice, and invertebrates, including oxidative stress, genotoxicity, inflammation, and reproductive toxicity, despite underrepresentation in the literature [57].

Polyethylene terephthalate (PET), with a Harmfulness Score of 0.6287, is widely distributed due to its use in textiles and packaging. Though often seen as benign, PET microplastics have been linked to cytotoxicity, gut dysbiosis, and pollutant adsorption, with growing health concerns tied to ingestion from bottled water. Biodegradation efforts using PETase and microbial remediation show promise but remain limited by environmental conditions [58]. Polymers such as polyamide (PA, Score = 0.6183), polyvinyl chloride (PVC, 0.6084), and polyurethane (PUR, 0.5979) present a moderate risk. PA disrupts endocrine and vascular systems [59], PVC leaches additives affecting soil and human health [60], and PUR causes aquatic toxicity and agricultural disruption, with resistance to degradation [61].

Biodegradable and lower‐emission plastics like polylactic acid (PLA, 0.5732), polycarbonate (PC, 0.5677), and polymethyl methacrylate (PMMA, 0.5559) are not without risk. PLA affects aquatic organisms and gut health [62], PC is found in household exposure routes and demonstrates liver toxicity [63], and PMMA contributes to endocrine and inflammatory responses, with high persistence due to its physicochemical structure [64].

The lowest‐ranking polymers, including acrylonitrile butadiene styrene [65] (ABS, 0.5308) polytetrafluoroethylene [66] (PTFE, 0.5201), ethylene‐vinyl acetate [67] (EVA, 0.5189), high‐[68] and low‐density [69] polyethylene (HDPE: 0.5026; LDPE: 0.4953), polyoxymethylene [70] (POM, 0.4844), polybutylene terephthalate [71] (PBT, 0.4679), and polyether ether ketone [72] (PEEK, 0.2289), still pose risks despite their lower scores. These include respiratory hazards from ultrafine particles (ABS, PTFE) [73], endocrine and developmental effects (EVA, HDPE, LDPE) [32], inflammation and pollutant adsorption (POM, PBT) [74], and potential ecological degradation (PEEK) [75].

Thus, the Harmfulness Score offers a nuanced and scalable method to rank microplastic polymers by environmental threat. It supports evidence‐based prioritization of mitigation and regulatory strategies by capturing not only experimental toxicity but also scientific consensus and exposure context. This framework can be directly employed by policymakers and researchers to design targeted interventions against microplastic pollution.

### 2.9 Environmental Risk Insights and Policy Implications

The Harmfulness Score confirmed polystyrene (PS: 0.7895), polyethylene (PE: 0.7710), and polypropylene (PP: 0.6632) as high‐risk polymers, aligning with empirical evidence of oxidative stress, genotoxicity, and enzymatic disruption. PS dominates marine environments (42.3%), while PE is prevalent in freshwater (38.7%) and terrestrial systems (27.9%), indicating distinct exposure pathways. PS impairs photosynthesis, development, and neurological function, whereas PE disrupts soil enzyme activity and causes histological damage in aquatic fauna, reinforcing the need for targeted, morphology‐sensitive mitigation strategies.

Microplastic–nanoparticle interactions, particularly involving TiO_2_, ZnO, and AgNPs, represent a critical area of emerging risk. Evidence supporting these risks derives mainly from controlled laboratory assays, complemented by limited field observations and modeling studies. While laboratory data provide strong mechanistic insight into oxidative stress, pollutant transport, and synergistic toxicity, extrapolation to ecosystems is constrained by variability in natural conditions. Field surveys confirm co‐occurrence but often lack mechanistic resolution, and predictive models remain dependent on sparse empirical inputs. This imbalance highlights both the plausibility and the uncertainty of current risk assessments, reinforcing the need for harmonized, long‐term monitoring frameworks. Their combined effects require integrated toxicological and functional assessment to inform predictive ecological models. Bio‐based alternatives such as PLA and PA, particularly when reinforced with graphene oxide or CNTs, are frequently promoted as sustainable options due to their potential for biodegradability and reduced carbon footprint. However, experimental evidence shows that their post‐use behavior and degradation are highly context‐dependent. PLA degrades most efficiently under industrial composting conditions, with slower rates in soil, wastewater, and aquatic environments where enzymatic activity and hydrolysis govern the process [76]. Reinforcing agents such as graphene oxide and CNTs can alter degradation pathways and rates, often reducing the overall biodegradability of the composites [77]. Life‐cycle assessments confirm that these materials may offer a lower carbon footprint than fossil‐based plastics, but outcomes vary depending on assumptions about end‐of‐life scenarios and waste management systems [78, 79]. Although industrial composting enables complete mineralization, real‐world degradation is typically slower, and discrepancies between laboratory and field results highlight the need for standardized protocols that mimic environmental conditions [80]. Moreover, PLA can fragment into microplastics faster than some petroleum‐based plastics, raising ecotoxicological concerns for aquatic and soil biota [81, 82]. These complexities demonstrate that while PLA and PA composites hold promise, they also demand rigorous post‐use evaluation to balance sustainability claims with actual environmental performance. Social factors, such as consumer preference for convenience (68%) and cost (57%), alongside limited behavioral change (24%), further compound risks, particularly where mismanagement leads to leakage rates up to 25%. This underscores the necessity for interdisciplinary governance linking materials science, regulation, and behavior change to ensure that bio‐based alternatives deliver genuine sustainability benefits.

We acknowledge that research coverage is skewed: PE and PS together account for nearly 18% of all documents, while polymers like PEEK appear in only 21 publications. To mitigate this imbalance, we incorporated a frequency‐based weight (𝑤_p_  =  0.7 + 0.3𝐹_p_) and a confidence function (σ_p_  =  1 − 1/(1 + 0.05𝑛_p_)) that penalizes low‐replication cases without discarding them. A skewness of ≈ 1.75 confirms that research output is unevenly distributed. Rare polymers retain ≥ 70% of their base score but are flagged as less certain, identifying research gaps rather than erasing them.

**FIGURE 5:**
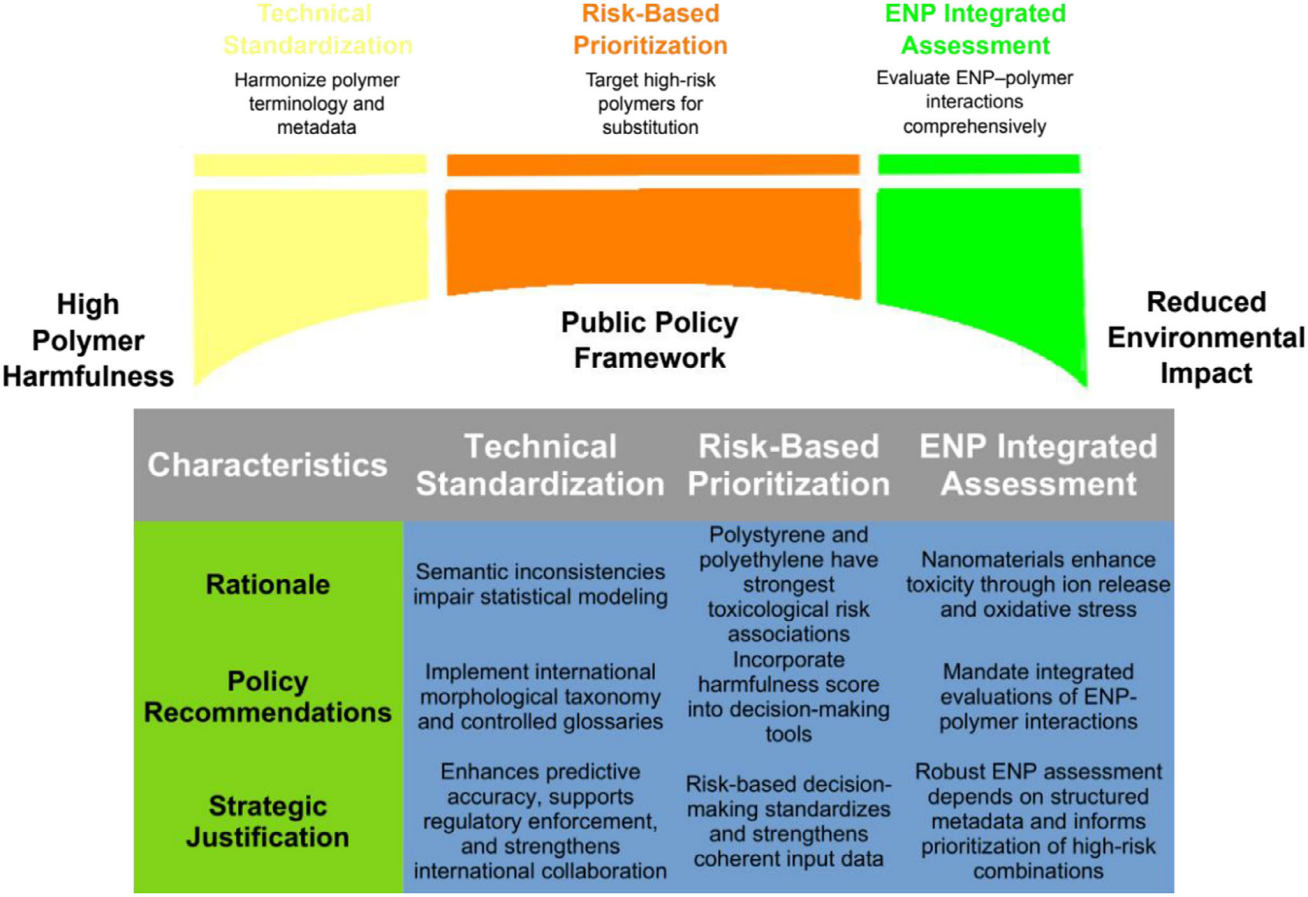
Proposed three‐axis public policy framework for mitigating microplastic and nanomaterial risks, structured around: (1) technical‐informational standardization of terminology and metadata, (2) risk‐based regulatory prioritization using the Harmfulness Score, and (3) integrated assessment of engineered nanoparticles (ENPs). Arrows indicate synergistic feedback among axes to support data‐driven and context‐sensitive implementation.

In response, we propose a three‐axis policy framework supported by the Harmfulness Score and visualized in Figure 5.

**Axis 1 focuses on technical–informational standardization**. Severe inconsistencies in polymer terminology (e.g., “fiber” vs “microfiber”) and the absence of essential descriptors such as particle size, density, or crystallinity—reported in fewer than 4% of abstracts—undermine data comparability. Recommendations include a globally harmonized morphological taxonomy, machine‐readable glossaries, metadata tagging, and integration with frameworks such as **ISO**, Darwin Core, and the Global Biodata Coalition [83, 84, 85, 86]. The feasibility of such harmonization is constrained by regulatory variability and diverse industrial practices, compounded by the complexity of microplastic size, shape, and chemistry. Nevertheless, examples from ISO, REACH, and Darwin Core show that collaboration, flexible frameworks, and the incorporation of scientific advances can drive alignment. Tools such as the MaTCH algorithm [87] and global laboratory networks [88] illustrate practical progress, though fragmented legislation [89] and inconsistent practices [14, 90] remain barriers. Hierarchical categorization [91] and community‐driven approaches [92] are promising, but success will ultimately depend on sustained collaboration and the alignment of regulatory frameworks with evolving scientific knowledge [93].

**Axis 2** centers on regulatory prioritization. Simulation studies show that targeted substitution of PS and PE could reduce ecotoxicological harm by up to 52% while affecting only 18–24% of applications, surpassing generic bans. Embedding the Harmfulness Score into mechanisms such as the Global Plastics Treaty and extended producer responsibility (EPR) systems is feasible but complex. Treaty negotiations emphasize life‐cycle management and substitution of harmful polymers [94, 95], and EPR frameworks incentivize sustainable materials [96]. More than 85% of countries support National Action Plans (NAPs), but their voluntary nature limits accountability [97]. Implementation faces political fragmentation [96, 98], industrial resistance without viable substitutes [99], and methodological challenges from chemical additives [100]. Policy innovation differentiated industrial measures, and public engagement can facilitate substitution [101], though success depends on political will, industrial adaptation, and the establishment of enforceable international frameworks.

**Axis 3** proposes the integrated evaluation of engineered nanoparticles (ENPs). ENPs can enhance toxicity via ion release and ROS generation, especially under UV or acidic conditions, underscoring the need for standardized assays, metadata, and integration with toxicological databases. Feasibility is supported by nanotoxicology initiatives that promote harmonized reporting, FAIR principles, and reference materials. Metadata lists such as NanoTox complement MIRIBEL, MINChar, and MIAN [102], while FAIR‐aligned interfaces enable cross‐domain reuse [103]. Standardization efforts include the International Standardisation Roadmap for Nanomedicine [104] and curated databases such as CoCoN [105]. Methodological advances, such as the new approach methodologies (NAMs) for next‐generation risk assessment [106] and tools like NanoDefiner [107], improve regulatory compliance and predictive capacity. Interlaboratory cytotoxicity comparisons [108] and advanced in vitro models [109] highlight reproducibility gains. Nonetheless, regulatory variability, nanomaterial complexity, and interdisciplinary gaps persist. Progress toward global harmonization will require adapting these lessons to ENP–polymer contexts to ensure consistent, reliable evaluations across regions and industries.

To ensure methodological impartiality, we conducted sensitivity tests varying XGBoost hyperparameters and confirmed stable rankings. Bootstrap resampling validated score robustness under document count uncertainty. In leave‐one‐out tests, removal of PE or PS did not lead to score inversion among mid‐ranked polymers, reinforcing the structural integrity of the metric.

Finally, the proposed policy axes will actively help address data gaps. Standardized metadata will improve representation of under‐studied polymers; embedding the score in regulatory tools will incentivize balanced reporting; and aligning ENP standards will enable niche polymers to accumulate sufficient coverage. Until these advances materialize, we must work transparently with the best available evidence.

Together, these three axes provide a robust, scalable strategy to mitigate the risks posed by microplastics and nanomaterials. Axis 1 ensures data quality, Axis 2 drives science‐based regulatory focus, and Axis 3 captures technological complexity. Their integration mirrors global initiatives such as the Chair's Text of the Global Plastics Treaty and aligns with the science‐policy vision outlined by Brooks and Havas [7]. Methodological transparency, cautious interpretation for low‐coverage polymers, and dynamic score updates are essential to preserve scientific fairness and policy relevance.

### 2.10 Recommendations

Mitigating micro‐ and nanoplastic risks requires a data‐driven approach built on three interconnected strategies. First, standardizing terminology and metadata through a globally aligned morphological taxonomy and interoperable glossaries—compatible with ISO, Darwin Core, and Global Biodata Coalition standards—addresses critical underreporting of particle characteristics such as size, density, and surface area, which are reported in fewer than 4% of abstracts yet are essential for accurate modeling and risk assessment. Second, integrating the Harmfulness Score into regulatory frameworks enables evidence‐based substitution of high‐risk polymers like polystyrene and polyethylene, reducing ecotoxicological impact while maintaining functionality across most applications. This prioritization was based primarily on toxicological risk, though we acknowledge that incorporating additional criteria such as economic feasibility, scalability, and consumer acceptance would strengthen future studies, and we encourage subsequent research to address these parameters. Third, consistent environmental assays are needed to evaluate polymer–nanoparticle interactions, particularly those involving TiO_2_, ZnO, and AgNPs. Detailed metadata on nanoparticle composition, dispersion, and encapsulation will improve reproducibility and guide the design of safer nanocomposites. These technical measures must be complemented by behavioral and infrastructural change, ensuring that scientific and regulatory advances translate into scalable, socially aligned environmental solutions.

## 3 Conclusions

In summary, a composite metric—Harmfulness Score—was developed and validated to quantitatively assess the environmental and biological risks associated with microplastic polymers. This score integrates sentiment polarity, impact keyword frequency, and degree centrality in scientific co‐occurrence networks to enable a comparative ranking of polymeric materials. Machine learning models, particularly XGBoost, were used to process over 100 000 abstracts vectorized by TF‐IDF and Bag‐of‐Words, incorporating additional features such as polymer type, morphology, and nanoparticle associations.

Polystyrene (PS) and polyethylene (PE) emerged as the most cited and toxicologically linked polymers, frequently associated with oxidative stress, cytotoxicity, and genotoxicity. In contrast, polymers such as PEEK, PLA, and PMMA appeared infrequently, highlighting significant asymmetries in research coverage. Morphological profiling revealed the dominance of fibers, films, and fragments, with size distribution modeling indicating multimodal profiles including subpopulations near the nanoplastic range. Geographic matrix analysis showed that polymers were often associated with nanoparticles such as TiO_2_, ZnO, and AgNPs for UV protection and antibacterial enhancement.

Toxicological co‐association matrices revealed that conventional polymers were overrepresented in connection with high‐risk terms like “bioaccumulation” and “genotoxicity”. Many articles failed to report key descriptors such as particle size, surface area, and density, limiting reproducibility and risk modeling. Network centrality correlated strongly with term frequency, supporting its inclusion in the Harmfulness Score.

Regression models using XGBoost demonstrated high predictive accuracy for the Harmfulness Score using variables like morphology, nanoparticle presence, and toxicological term co‐occurrence. Final score adjustments accounted for citation‐based confidence and frequency weighting. The validation process confirmed a research bias toward a few polymers and an average confidence level of 0.82 across materials.

Overall, this study provides a scalable and interpretable method for ranking microplastic polymers by their environmental and biological impact, supported by bibliometric, morphological, and toxicological evidence. All models, datasets, and visualizations were stored in interoperable formats to enable reproducibility and policy benchmarking.

## Author Contributions

**Fernando Gomes**: Conceptualization, Writing—original draft, Funding, Programming. **Thomas Thundat**: Supervision, Writing—review & editing. **Shekhar Bhansali**: Supervision, Writing—review & editing.

## Conflicts of Interest

The authors declare no conflicts of interest.

## Declaration of Generative AI and AI‐Assisted Technologies in the Writing Process

Generative **AI** tools such as ChatGPT‐4o, Claude Sonnet 3.7, and Grammarly were employed to improve grammar, clarity, and tone in support of non‐native English speakers. The authors affirm that all AI‐assisted content was rigorously reviewed and that they remain fully accountable for the accuracy and integrity of this work.

## Supporting information


**Supporting File**: marc70094‐sup‐0001‐SuppMat.docx.

## Data Availability

The data that support the findings of this study are available from the corresponding author upon reasonable request.
