# Supplementary material for: Harmfulness Score: A Data‐Driven Framework for Ranking Environmental Risks of Microplastics
Source: Macromol Rapid Commun. 2025 Oct 21;46(24):e00559. doi: 10.1002/marc.202500559 (PMC12713616; doi:10.1002/marc.202500559)
Supplement: Supplementary file 1 — Supporting File: marc70094‐sup‐0001‐SuppMat.docx. [file MARC-46-e00559-s001.docx]

**Harmfulness Score: A Data-Driven Framework for Ranking Environmental Risks of Microplastics**

Fernando Gomes S. Jr.^[a,b,c]^*, Shekhar Bhansali ^[d]^ **and Thomas Thundat^[e]^**

_____________________________________________________________________

[a] Prof. Dr. Fernando Gomes S. Jr.

Instituto de Macromoléculas Professora Eloisa Mano

Universidade Federal do Rio de Janeiro

Centro de Tecnologia – Cidade Universitária

Rio de Janeiro, 21941-598 (Brazil)

E-mail: fgsj@ufrj.br*

[b] Prof. Dr. Fernando Gomes S. Jr.

Programa de Engenharia da Nanotecnologia – COPPE

Universidade Federal do Rio de Janeiro

Centro de Tecnologia – Cidade Universitária

Rio de Janeiro, 21941-972 (Brazil)

E-mail: fgomes@pent.coppe.ufrj.br

[c] Prof. Dr. Fernando Gomes S. Jr.

Department of Electrical and Computer Engineering

Florida International University

10555 West Flagler Street, EC3900

Miami, FL 33174 (USA)

E-mail: fgomesde@fiu.edu

[d] Prof. Dr. Shekhar Bhansali

Dept. of Electrical and Computer Engineering

Vanderbilt University

Featheringill Hall, 2301 Vanderbilt Place

Nashville, TN 37235 (USA)

E-mail: shekhar.bhansali@vanderbilt.edu

[e] Prof. Dr. Thomas Thundat

Department of Chemical and Biological Engineering

University at Buffalo – The State University of New York

Buffalo, NY 14260 (USA)

**E-mail: tgthunda@buffalo.edu**

# **Supplementary Information – Introduction**

**This Supplementary Information accompanies the article** “Harmfulness Score: A Quantitative Approach to Assessing Microplastics Environmental Impact” **by Fernando Gomes S. Jr., Shekhar Bhansali, and Thomas Thundat.**

It provides a comprehensive account of the computational methodology, data processing pipelines, and analytical frameworks used to construct the Harmfulness Score—a composite metric designed to rank microplastic polymers by environmental and toxicological risk. The contents include:

- Details of the systematic literature search and filtering criteria applied to 104,471 scientific abstracts retrieved from Scopus.
- Description of data merging, standardization, and preprocessing steps for bibliometric and morphological analysis.
- Network construction procedures using VOSDataAnalyzer and NetworkX, including cluster analysis, term centrality calculations, and trend evaluation by publication year.
- Quantitative modeling of particle size distributions, morphological descriptors, and co-occurrence heatmaps linking polymers to engineered nanoparticles and impact categories.
- Machine learning implementation using Random Forest and XGBoost, with preprocessing via TF-IDF and SVD, and supervised classification of sentiment polarity.
- Full derivation of the Harmfulness Score formula, incorporating sentiment, impact frequency, network centrality, frequency weight, and statistical confidence.
- Diagnostic procedures to assess model robustness, publication skewness, and variable importance in risk prediction.

All data handling scripts, preprocessing protocols, and additional figures referenced in the main article are described or outlined here to ensure reproducibility and transparency.

## Literature Search and Data Collection

A systematic literature search was conducted using the Scopus database to gather research publications pertinent to microplastics and nanoplastics. The primary goal was to comprehensively identify trends, methodologies, and knowledge gaps within this research domain. The search query was crafted to capture a wide range of relevant studies by including multiple variations of the terms “microplastic” and “nanoplastic.” Specifically, the query used was:

ALL("microplastic" OR "micro-plastic" OR "nanoplastic*" OR "nano-plastic*")

The initial search, conducted without restrictions on publication year, yielded 104,471 documents on March 1, 2025. To facilitate a structured investigation and enable trend analysis, the dataset was segmented into distinct time periods. The historical literature, spanning from 1961 to 2020, was compiled separately, while publications from 2021 to 2025 were treated as independent datasets. Given the substantial volume of data, records were stored as individual CSV files corresponding to each time period, systematically named (e.g., scopus1961-2020.csv, scopus2021.csv, scopus2022.csv, etc.), with some years requiring multiple partitions due to the volume of records.

To ensure the relevance and quality of the collected literature, a set of filtering criteria was applied. Only peer-reviewed journal articles and review papers were retained, while conference proceedings, book chapters, and editorials were excluded. Additionally, specific subject areas, such as chemistry, were omitted in certain searches to prioritize research focused on the environmental and biological aspects of microplastics and nanoplastics. The dataset segmentation by publication year enabled a detailed temporal trend analysis. The final dataset, encompassing multiple CSV files, contains metadata for each document, including the title, authorship, source, year of publication, and citation count. The total volume of collected data amounts to 1.9 GB, underscoring the breadth and depth of research literature in this field and providing a robust foundation for further analysis.

## Data Merging and Preprocessing

Following data collection, the exported CSV files were merged into a single dataset and converted into the Parquet format to optimize storage and enable efficient querying. This was achieved using a custom Python script, CSVMerge_to_parquet_format.py, which automated the process by scanning the working directory for relevant CSV files, loading them into Pandas DataFrames, and concatenating them into a unified dataset. The final dataset was stored as microplastic_analysis_filtered.parquet using the Snappy compression algorithm, significantly enhancing storage efficiency and reducing query execution time, thereby improving the scalability of subsequent analyses.

## Network Analysis

To extract relationships and research trends, network analysis was conducted using the VOSDataAnalyzer v0.3 software, which processes VOSviewer MAP and NET files. The analytical pipeline involved multiple stages, beginning with data input and preprocessing. The software’s graphical user interface (tkinter) allowed users to upload MAP and NET files, and any missing dependencies, such as NumPy, pandas, seaborn, Matplotlib, and Plotly, were automatically installed. The dataset was subsequently cleaned and sorted according to cluster classification and Total Link Strength (TLS), ensuring that the network structure was appropriately represented.

A statistical analysis was conducted to examine the correlation between TLS and the number of occurrences of specific terms. A scatter plot of TLS versus Occurrences was generated, and a linear regression model was fitted to the data. The coefficient of determination (R²) was calculated to assess the correlation strength, and a new column, TLS_per_Occ, was computed to represent the ratio of Total Link Strength to Occurrences. These calculations provided a quantitative measure of term connectivity within the network, indicating the relative importance of specific terms.

To refine cluster analysis, terms were ranked based on two criteria: the most relevant nodes per cluster, determined by their frequency of occurrence, and the most recent nodes, assessed based on their average publication year. These analyses enabled the identification of well-established research themes as well as emerging topics. The dataset was further sorted to prioritize clusters containing the most recent literature, allowing a temporal evaluation of trends within the research field.

For visualization and reporting, treemaps were generated using Plotly Express, highlighting the top five terms per cluster based on occurrences and the most recent terms per cluster based on publication year. Additionally, bar charts were created to depict term distributions across clusters, and a Tukey analysis was performed to assess statistically significant differences in publication year trends across research clusters. The network structure was further examined using graph-based visualizations, in which Euclidean distance calculations were applied to assess the similarity between terms. A final DOCX report was generated, summarizing the correlation between Total Link Strength and Occurrences, and conditional formatting was applied to Excel outputs to enhance readability. Processed data was exported in multiple formats, including CSV, Excel, PNG, and HTML, ensuring accessibility for further research applications.

## Bibliometric and Morphological Analysis Methodology

### **Dataset and Preprocessing**

This phase of the study was based on an extensive corpus of over 100,000 scientific articles related to microplastics, stored in Parquet format to optimize both storage efficiency and query performance. Each record contained structured metadata, including titles, abstracts, publication years, countries of origin, polymer types, particle sizes and shapes, analytical techniques, nanoparticle associations, and reported biological or environmental impacts.

To ensure data integrity and consistency, a comprehensive cleaning and standardization procedure was performed. All string values were lowercased, stripped of punctuation, and normalized using custom dictionaries to harmonize naming conventions. This was particularly critical for semicolon-separated fields, such as those listing multiple polymers, nanoparticles, or impacts. For instance, nanoparticle designations like “TiO2 NP” and “Titanium Dioxide” were standardized to “TiO₂,” ensuring coherence in downstream analysis.

### **Frequency and Co-occurrence Analysis**

The cleaned dataset was subjected to frequency analysis to determine the prevalence of polymers, particle shapes and sizes, nanoparticles, environmental impact descriptors, and associated methodologies. For multi-valued fields, semicolon-delimited values were split and individually counted to capture all relevant instances. To investigate relationships between different categories, co-occurrence matrices were generated. These included associations between polymer types and nanoparticles, particle shapes, environmental impacts, and analytical techniques. The relationships were quantified using group-by aggregation and unstacking functions in Pandas, and the resulting matrices were visualized using Seaborn to generate heatmaps with and without annotations.

### **Particle Size Distribution Modeling**

To analyze the distribution of particle sizes within the dataset, a non-linear regression model was applied to develop a probability density function. The particle size values, extracted from numeric entries between 0 and 5 mm, were fitted with four overlapping distribution curves, each corresponding to a distinct modal region. The area under each component curve was calculated using the trapezoidal rule. This modeling approach allowed the characterization of the polydispersity of microplastics and revealed the presence of distinct particle size subpopulations, including ranges that border on nanoplastics.

### **Morphological Distribution Analysis**

A morphological mapping analysis was conducted to assess the distribution of particle shapes across the most commonly studied polymers. Shape descriptors such as “fiber,” “film,” “fragment,” “foam,” and “particle” were harmonized through label mapping. A matrix was constructed to summarize the frequency with which each polymer appeared in conjunction with each shape category. This enabled the identification of degradation trends, revealing how different polymers fragment and transform in environmental contexts.

### **Nanoparticle–Polymer Interaction Analysis**

To examine the scientific and technological linkages between polymers and engineered nanoparticles, co-occurrence matrices were built based on shared appearance in articles. The dataset included key nanoparticles such as TiO₂, ZnO, AgNPs, graphene oxide (GO), and carbon nanotubes (CNTs). The resulting heatmaps highlighted patterns of material pairing, such as the frequent co-use of TiO₂ with polyethylene for UV stabilization or antimicrobial enhancement. These insights offered clues into emerging research synergies and potential application domains.

### **Environmental and Toxicological Association Mapping**

To understand the environmental and biological implications of various microplastic materials, two additional matrices were developed. The first captured the frequency with which specific polymers were associated with toxicological terms such as “bioaccumulation,” “cytotoxicity,” “oxidative stress,” and “genotoxicity.” The second matrix analyzed similar associations for nanoparticles. These co-occurrence patterns provided insight into which materials were most commonly studied in the context of environmental risk, helping to map the distribution of concern across the scientific literature.

### **Geographic Distribution Analysis**

Geographic trends in microplastics research were explored by extracting country names from metadata fields. When multiple countries were listed in a single entry, they were separated and normalized. Co-occurrence matrices were created to relate countries to polymers and nanoparticles studied in their research output. Heatmaps of these matrices revealed regional research priorities.

### Machine Learning and Harmfulness Modeling Methodology

This study employed a comprehensive machine learning and text mining framework to evaluate sentiment, quantify harmfulness, and analyze structural relationships in the scientific literature on microplastics. Two supervised learning models—Random Forest and XGBoost—were used for classification and regression tasks, leveraging their respective strengths in handling structured and unstructured data. The methodological pipeline included data preprocessing, feature engineering, unsupervised clustering, network analysis, model training, and the construction of a composite harmfulness score.

#### **Data Preparation and Text Processing**

The dataset was composed of scientific articles in .parquet format, including metadata, titles, and abstracts. Textual data underwent cleaning procedures such as lowercasing, punctuation and digit removal, and whitespace normalization. Two vectorization techniques—Term Frequency–Inverse Document Frequency (TF-IDF) and Bag-of-Words (BoW)—were then applied to numerically encode the text. These representations provided the basis for clustering, sentiment analysis, and subsequent modeling.

#### **Feature Engineering**

To incorporate structured data into the models, categorical variables—including polymer type, chemical composition, particle size, morphology, associated nanoparticles, and environmental impact descriptors—were transformed using label encoding and one-hot encoding, depending on the variable type. To reduce feature dimensionality while retaining critical semantic patterns, Principal Component Analysis (PCA) and Truncated Singular Value Decomposition (SVD) were employed.

#### **Clustering and Thematic Analysis**

Unsupervised learning was used to uncover dominant research topics within the dataset. The KMeans algorithm segmented the corpus into 14 empirically optimized clusters, based on semantic similarity in the reduced-dimensionality space. In parallel, cosine similarity was used to assess inter-document relationships. These cluster assignments and similarity scores were retained as contextual metadata for downstream modeling and interpretation.

#### **Sentiment Analysis and Classification Modeling**

The VADER Sentiment Analyzer was applied to article abstracts to extract sentiment polarity scores. Based on compound polarity thresholds, each document was labeled as expressing positive, neutral, or negative sentiment toward microplastic impacts. These sentiment classes were used as target variables for Random Forest Classifier and XGBoost Classifier models, which were trained using both text-derived and structured features.

Model evaluation employed standard classification metrics, including accuracy, precision, recall, and F1-score. Hyperparameter optimization was conducted using RandomizedSearchCV, and Recursive Feature Elimination with Cross-Validation (RFECV) was used to identify the most predictive variables.

#### **Network Analysis and Integration**

To better understand the contextual importance of polymers and their interactions with other variables, a co-occurrence network was constructed using NetworkX. In this network, nodes represented polymers, nanoparticles, morphological descriptors, and environmental outcomes, while edges represented co-occurrence within the same study. Key network metrics—including degree centrality, eigenvector centrality, and betweenness centrality—were computed to identify structurally significant materials and themes. These centrality values were later incorporated into the composite harmfulness assessment.

#### **Visualization and Reporting**

All intermediate and final outputs were stored in structured formats including CSV, Excel, Markdown, and HTML to ensure accessibility. Visualization outputs included heatmaps, network diagrams, and bar charts showing feature importance and classification performance. These visual tools facilitated both internal analysis and external communication of the findings.

#### **Harmfulness Score Calculation**

**The Harmfulness Score for microplastic polymers is computed through a multi-step formulation that integrates sentiment polarity, impact severity, and network centrality. These components are aggregated into a base score using normalized and weighted values, and subsequently adjusted by a frequency-based factor and a confidence metric based on citation count.**

**The sentiment component**
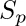
**is obtained by normalizing the absolute value of the compound polarity score produced by the VADER sentiment analyzer, ensuring that higher negativity (more harmful perception) corresponds to higher values. This is defined as:**


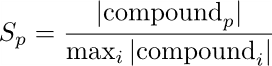
 **(1)**

**The impact component**
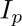
 **is calculated as the fraction of environmentally harmful keyword occurrences relative to the total number of impact-related mentions in the text. It is first computed as a raw proportion:**


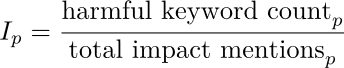
 **(2)**

**and then normalized across all polymers for comparability:**


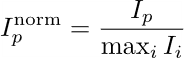
 **(3)**

**The centrality component**
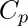
**, which reflects the structural prominence of a polymer within a co-occurrence network of interactions (e.g., with nanoparticles or environmental outcomes), is defined using degree centrality in this execution. It is normalized as follows:**


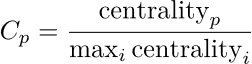
 **(4)**

**These three components are linearly combined to form the base harmfulness score, with empirically chosen weights favoring sentiment and impact equally, and assigning a smaller contribution to centrality:**


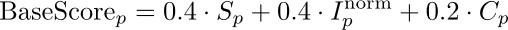
 **(5)**

**To adjust for how frequently a polymer appears in the dataset, a relative frequency weight**
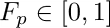
 **is computed by dividing the number of documents**
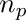
 **in which the polymer appears by the maximum document count among all polymers:**


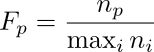
 **(6)**

**This is then incorporated into a frequency weight function that prevents rare polymers from being completely disregarded:**


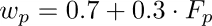
 **(7)**

**In parallel, a confidence score**
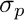
 **is computed as a logistic function that asymptotically approaches 1 for well-documented polymers. This accounts for statistical reliability and penalizes polymers with low representation:**


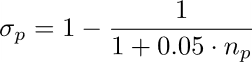
 **(8)**

**Finally, the overall harmfulness score for each polymer**
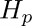
 **is calculated by multiplying the base score by the frequency weight and the confidence score:**


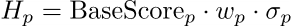
 **(9)**

**This formulation ensures that polymers with strong negative sentiment, frequent mentions of harmful impacts, and central roles in co-occurrence networks receive proportionally higher scores, while accounting for statistical confidence and document prevalence.**

**While this formulation incorporates safeguards to reduce statistical noise and mitigate distortions from coverage imbalance, the potential for reporting bias remains a known limitation. The frequency-based weight preserves at least 70 % of the base score even for under-represented polymers, and the confidence function adjusts the final score according to citation volume. However, the observed skewness in research distribution—where a few polymers such as PE and PS dominate the literature—can still amplify their relative influence. These limitations underscore the need for cautious interpretation of scores for sparsely studied polymers. Future improvements in data availability, reporting standards, and metadata quality will be essential to ensure increasingly fair and comprehensive assessments. Until then, this framework provides a transparent and adaptive starting point based on the best available evidence.**

**To systematically examine whether the Harmfulness Score remains valid under a ‘no bias in reporting’ assumption, we conducted a four-part analysis implemented in the final step of the computational pipeline. First, we calculated the skewness of publication counts per polymer to evaluate the symmetry of research distribution; a skewness of ≈ 1.75 indicated significant concentration around a few polymers, suggesting bias. Second, we computed the average confidence score across all polymers (≈ 0.82), which quantifies reliability based on citation volume. Third, we identified the top 25 % most harmful polymers based on the final score, flagging them as priority targets. Lastly, we listed the bottom 25 % in terms of publication volume, highlighting under-studied materials. This diagnostic procedure ensures that the model not only ranks harmful polymers but also transparently communicates where the supporting evidence is weakest and where future research is most needed.**

To explore how polymer characteristics relate to their Harmfulness Scores, we trained regression models using Random Forest and XGBoost on structured features. These models were evaluated using an 80/20 training-test split and assessed via R^2^, MSE, and RMSE. **Feature importance analysis revealed the variables most strongly influencing the predicted risk rankings, providing interpretability and reinforcing the scientific relevance of the score.**
